# PREVALENCE AND PREDICTORS OF LONELINESS AMONG CAREGIVERS BY CARE RECIPIENT’S LEVEL OF COGNITIVE IMPAIRMENT

**DOI:** 10.1093/geroni/igae098.3693

**Published:** 2024-12-31

**Authors:** Elyse Couch, Rebecca Howe, Jodi L Southerland

**Affiliations:** Brown University, Providence, Rhode Island, United States; VA Providence Healthcare System, Providence, Rhode Island, United States; East Tennessee State University, Johnson City, Tennessee, United States

## Abstract

Loneliness is increasingly being recognized as a public health issue. However, little is known about the prevalence or predictors of loneliness among caregivers in the United States (US). This study aimed to assess the prevalence and predictors of loneliness among US caregivers by the care recipient’s level of cognitive impairment. We conducted a cross-sectional study of caregivers for Medicare beneficiaries in the US using data from the National Study of Caregiving (NSOC). Caregivers were divided into three groups by the care recipient’s level of cognitive impairment: non-dementia, MCI, and ADRD caregivers. Logistic regression models were used to identify predictors of severe loneliness (defined as feeling lonely every day or most days) among caregivers. Sample weights were applied to all analyses. Overall, 8.5% of caregivers reported feelings of severe loneliness, equating to over 750,000 caregivers when sample weights were applied. The prevalence of severe loneliness was higher in dementia caregivers (13%) compared to MCI (7.4%) and non-dementia (7.1%) caregivers. Adjusted logistic regression models found dementia caregiving, being without a current partner, and reporting emotional difficulties with the caregiving role were significantly associated with severe loneliness. Increasing age and caring for a parent/other relative (vs spouse) were associated with reduced odds of loneliness. There was no difference in odds of severe loneliness by gender or race. Dementia caregiving is associated with an increased risk of loneliness. Future research is needed to understand how loneliness affects caregiver health and wellbeing outcomes and to develop interventions to address caregiver loneliness, particularly for dementia caregivers.

